# The Effect of α-Mangostin and Cisplatin on Ovarian Cancer Cells and the Microenvironment

**DOI:** 10.3390/biomedicines10051116

**Published:** 2022-05-11

**Authors:** Paulina Borzdziłowska, Ilona Bednarek

**Affiliations:** Department of Biotechnology and Genetic Engineering, Faculty of Pharmaceutical Sciences in Sosnowiec, Medical University of Silesia, 40-055 Katowice, Poland; ibednarek@sum.edu.pl

**Keywords:** exosomes, α-mangostin, cisplatin, ovarian cancer, NTA—nanoparticle analysis tracking

## Abstract

Ovarian cancer is one of the cancers that, unfortunately, is detected at a late stage of development. The current use of treatment has many side effects. Notably, up to 20% of patients show cisplatin resistance. We assess the effects of cisplatin and/or α-mangostin, a natural plant derivative, on ovarian cancer cells and on the cancer cell microenvironment. The effect of cisplatin and/or α-mangostin on the following cells of ovarian cancer lines: A2780, TOV-21G, and SKOV-3 was verified using the XTT cytotoxicity assay. The separate and combined effects of tested drugs on ovarian cancer cell viability were assessed. We assessed the influence of chemotherapeutic agents on the possibility of modulating the microenvironment. For this purpose, we isolated exosomes from drug-treated and untreated ovarian cancer cells. We estimated the differences in the amounts of exosomes released from cancer cells (NTA technique). We also examined the effects of isolated exosome fractions on normal human cells (NHDF human fibroblast line). In the present study, we demonstrate that treatment of A2780, SKOV-3, and TOV-21G cells with α-mangostin in combination with cisplatin can allow a reduction in cisplatin concentration while maintaining the same cytotoxic effect. Ovarian cancer cells release a variable number of exosomes into the microenvironment when exposed to α-mangostin and/or cisplatin. However, it is important to note that the cargo carried by exosomes released from drug-treated cells may be significantly different.

## 1. Introduction

The cell’s microenvironment is created by factors that directly influence the conditions around the cells. For a single cell, the term “microenvironment” applies to the following: the extracellular matrix (ECM), the surrounding cells of the same or different type; hormones, cytokines, and other bioactive molecules released via an autocrine, endocrine, or paracrine pathway; in other words, nano changes in the function and structure of ECM molecules and mechanical changes caused by body movement or body fluids [1,2,3]. All these factors have an impact on a single cell and either promote or inhibit its growth and development. The task of the microenvironment is to maintain a balance between proliferating and differentiating cells [4]. This term refers not only to normal cells but also to tumor cells. The neoplastic microenvironment includes the following: fibroblasts, immune cells, and endothelial cells. Cells and the microenvironment elements surrounding them can influence the changes taking place in the tumor microenvironment (TME). Changes in the TME cover all stages of cancer development—from initiation, progression, and metastasis to invasion. Research has confirmed that exosomes are a factor that affects cancer cells themselves and the elements making up the TME. Certain groups of scientists examine the exosomes’ share in cancer progression regarding their origin and the severity of the disease [5,6,7].

The term “exosome” was introduced by Rose M. Johnstone in 1987. The exosome can be referred to as a vesicular molecule secreted by the multi-vesicle corpuscle (MVB) [8]. Over the following years, numerous studies on the origin and structure of exosomes have been undertaken and conducted. Many questions about exosomes have remained unanswered so far. However, recently, numerous studies have been conducted to find these answers [9].

Exosomes from different cells are secreted into the surrounding environment. Their physiological role varies depending on the body fluid in which they are located. Thus, exosomes are present in urine, saliva, synovial fluid, bile, cerebrospinal fluid, nasal fluid, uterine fluid, amniotic fluid, mother’s milk, blood, feces, or semen [10,11]. Exosomes, which are part of the microenvironment, play an essential role both in physiological and cancerous conditions. Wang et al. [12] identified three primary mechanisms of exosome activity related to the development of cancer, i.e., as follows:

1. Interaction between exosomes and cells of the immune system;
2. Influence of exosomes by delivering a molecular charge in the form of miRNA, mRNA, DNA fragments, and proteins;
3. Participation of exosomes in the processes of invasion, metastasis, and drug resistance, acting as communication signals between cells [12].

Ovarian neoplasms are a large group of heterogeneous tumors. Their classification is not simple, and it is based on various factors of diagnostic importance and disease prognosis. There are currently two major ratings of ovarian cancer, developed independently by WHO and FIGO (International Federation of Gynecology and Obstetrics). The FIGO classification is based on the cancer stage, while the WHO classification is based on the histopathological and molecular tumor types. Both of these aspects are essential criteria in the selection of basic, differentiated therapy [13,14,15].

Early-stage ovarian cancer is very rarely detected. That is why it is still called “the silent killer”. Proper and effective screening methods need to be developed. Screening would significantly reduce the mortality rate among patients with an early diagnosis of ovarian cancer. [16].

In most cases, systemic chemotherapy is indicated after surgery. Platinum derivatives and taxanes are used as basic drugs, most often in combination therapy. The following two treatment regimens can be used: cisplatin with paclitaxel or carboplatin with paclitaxel. The efficacy of both regimens is comparable, with carboplatin being administered more easily to the patient and generally better tolerated by their body [17].

Difficulties in the treatment of patients with ovarian cancer result from late diagnosis and currently used treatment regimens. With this in mind, it is important to understand the exact development mechanism of ovarian cancer, including the formation of metastases, in which exosomes are involved.

Studies on the search for natural anticancer substances are still ongoing worldwide. Research on the possibility of using new substances in chemotherapy is underway. One such compound is α-mangostin from Garcinia mangostana Linn. [18,19,20,21]. It is a compound belonging to the group of xanthones, which shows a broad spectrum of activity, including anticancer [22,23,24,25,26]. Interest has been aroused due to the fact that mangosteen-derived xanthones not only stop cell division but also lead to cell death through the formation of apoptotic bodies, condensation, and fragmentation of the cell nucleus. Xanthones have antiproliferative properties as well as the ability to induce apoptosis. The exact mechanism of α-mangostin activation of apoptosis has been investigated in cancer cells of various lines. An increase in the concentration of caspase 9, caspase 8, and, most importantly, caspase 3 has been proven [27,28]. However, elevated levels of caspase 8 have not been observed in the HL-60 line (human acute promyelocytic leukemia cell line). In such cells, a slight increase in the activity of caspase 2 has been observed, which probably contributes to the enhancement of the apoptotic signaling pathway. The mitochondrial membrane potential being disturbed by α-mangostin is, in turn, evidenced by the increased level of cytochrome c in cells. The accumulation of reactive oxygen species and a decreased level of intracellular ATP have been registered in cells [29,30]. ATPase inhibition also significantly contributes to the stimulation of the apoptotic pathway [31]. The induction occurs at a level of regulation of proteins from the BCL-2 family. The expression of the FAS, BID, t-BID genes, p53, phosphorylated p53, and proteins BMF and BAX changes under the influence of α-mangostin. The last one is directly responsible for the membrane damage and the release of cytochrome c [32]. In addition, α-mangostin may act through the caspase-independent pathway, contributing to apoptosis [33].

An important feature of α-mangostin is its potential inhibitory effect on the formation of metastases. It blocks both migration and invasion of neoplastic cells through various molecular pathways, including, for example, the inactivation of JNK (c-Jun N-terminal kinases), the inhibition of NF-κB (Nuclear factor kappa-light-chain-enhancer of the activated B cells), c-Fos, and c-Jun, therefore contributing to the reduction of activity of MMP-2 and MMP-9 [34,35].

Last but not least, a significant aspect of the anticancer activity of α-mangostin is the interaction with anticancer drugs already widely used in medicine. A lot of research has been devoted to the impact that various substances of natural origin have on the effectiveness as well as the alleviation of the side effects of naturally obtained drugs in general. A synergistic effect has been observed for α-mangostin with 5-fluorouracil, which leads to the enhancement of the cell cycle inhibition of treated cells. Such results give new hopes and possibilities for using α-mangostin in anticancer therapy [36,37,38].

The aim of the study was to evaluate in vitro the cytotoxicity of various compounds on ovarian cancer cells of various origins. Cisplatin (currently used chemotherapeutic agent) and α-mangostin (a natural plant derivative) were used in the research. The drugs were tested separately and in the following combination: cisplatin and α-mangostin. This study focuses on the possibility of using α-mangostin alone or in combination with cisplatin in ovarian cancer chemotherapy. In addition, an indirect evaluation of the drug’s effects on the microenvironment of ovarian cancer cells was performed. The change in the amount of exosomes released by the cancer cells was assessed after treatment with α-mangostin and/or cisplatin. Additionally, an attempt was made to answer whether exosomes secreted from tumor cells show a different effect on normal fibroblasts after drug stimulation.

## 2. Materials and Methods

### 2.1. Cell Culture

Three human ovarian cancer lines and one fibroblast line were used in the research. SKOV-3 human ovarian cancer (adenocarcinoma) cells from American Type Culture Collection (ATCC^®^ HTB-77™) were cultured in RPMI-1640 medium with 2 mM L-glutamine (Lonza, Bazylea, Switzerland) supplemented with 10% FBS (fetal bovine serum, Gibco, Waltham, MA, USA) and 50 µg/mL gentamycin (Sigma-Aldrich/Merck, Saint Louis, MI, USA). TOV-21G human ovarian cancer (grade 3, stage III, primary malignant adenocarcinoma; clear cell carcinoma) cells from American Type Culture Collection (ATCC^®^ CRL-11730™) were grown in a mixture (1:1) of MCDB-105 medium (Biological Industries, Kibbutz Beit-Haemek, Israel), and M-199 Earle’s Salts Base medium (Biological Industries, Kibbutz Beit-Haemek, Israel) supplemented with 15% FBS and 50 µg/mL gentamycin. A2780 human ovarian cancer cells from the European Collection of Authenticated Cell Cultures (ECACC 93112519) were cultured in RPMI-1640 medium with 2 mM L-glutamine supplemented with 10% FBS and 50 µg/mL gentamycin.

NHDF Normal Human Dermal Fibroblasts from PromoCell bank (C-12300) were cultured in FBM Medium (Fibroblast Growth Basal Medium, Lonza, Bazylea, Switzerland) supplemented with 10% FBS with the addition of FGM™-2 Fibroblast Growth Medium-2 SingleQuots™ Supplements and Growth Factors, (it is insulin and fibroblast growth factor Lonza, Bazylea, Switzerland), and 50 µg/mL gentamycin.

Before isolation of exosomes, cells of all lines were cultured in a medium containing Exosome-Depleted FBS (dFBS, Gibco, Waltham, MA, USA). Cell lines were cultivated at 37 °C in a humidified atmosphere of 95% air and 5% CO_2_.

### 2.2. Drugs

Two compounds were used in the study. Alpha-Mangostin from Sigma-Aldrich (M3824) dissolved in methanol at a concentration of 24 mM stored at −20 °C. Cisplatin from Sigma-Aldrich (P4394), dissolved in 0.9% NaCl solution at a 1 mg/mL concentration, stored at −20 °C.

### 2.3. Cell Viability Assay

The XTT-based assay was used in the cell lines tested to determine IC50 values for α-mangostin and/or cisplatin. The determined IC50 value corresponds to the concentration of the test compound that leads to a 50% inhibition of cell proliferation in in vitro culture.

The assay was performed using Cell Proliferation Kit II (XTT: 2,3-Bis(2-methoxy-4-nitro-5-sulfophenyl)-2H-tetrazolium-5-carboxanilide inner salt dissolved in PBS—phosphate-buffered saline solution; Roche, Bazylea, Switzerland).

Prior to drug stimulation, cells were synchronized (maintained for 24 h in culture medium without FBS after seeding). After synchronization, the next steps were proceeded. Cells were seeded in 96-well plates, grown overnight, and treated with α-mangostin and/or cisplatin for 24 h at the right concentration (3.125–200 µM). To carry out the test, 100 µL of medium (RMPI-1640 or mixture (1:1) of MCDB-105 medium and M-199 Earle’s Salts Base medium) without phenol red containing the XTT substrate (0.2 mg/mL) and reaction activator PMS (phenazine methosulfate, 2 µg/mL) was added into each well of 96-well plates. Plates were incubated at 37 °C for 3 h in the dark. The absorbance of each well was measured at 450 nm with an ELISA plate reader (Dynex Technologies Triad Multi-Mode Microplate Reader, Thermo Fisher Scientific, Waltham, MA, USA). Controls (100% viable) were untreated cells of the respective test ovarian cancer line subjected to the XTT assay procedure. The obtained absorbance level for untreated cells was taken as 100% viability.

Relative IC50 values were determined for each drug and each ovarian cell line. The XTT test procedure was performed according to the instructions for A2780, SKOV-3, and TOV-21G cells treated with α-mangostin or cisplatin. The obtained absorbance values were related to the control (untreated cells of the respective cell line), which allowed us to determine the cell survival for a given tested drug concentration. Samples were prepared in triplicates. On the basis of the obtained results, a curve of the dependence of the concentration of the investigated drug (α-mangostin or cisplatin) on the cell viability was prepared.

### 2.4. Drug Combination Studies

The evaluation of the combined effect of α-mangostin with cisplatin on cell viability was performed exactly as described for the XTT assays. Drugs were combined in inconsistent proportions. Each concentration of α-mangostin was mixed with each concentration of cisplatin. The nature of the interaction between α-mangostin and cisplatin was assessed by concurrent treatment with both drugs for 24 h. The measure of synergy between the two drugs, called the combination index (CI), was calculated using the CompuSyn software (ComboSyn, Inc., Cambridge, MA, USA) developed on the basis of the mathematical algorithm of the median effect [39]. A drug combination was assumed to act synergistically if its CI was below 0.9; the combination was additive when the CI ranged from 0.9 to 1.1; the combination was antagonistic, as indicated by CI values above 1.1 [40].

### 2.5. Treatment of Cells and Isolation of Exosomes

A2780, TOV-21, and SKOV-3 cells were grown in 75 cm^3^ flasks. Based on the cytotoxicity results obtained, cells were treated with α-mangostin and/or cisplatin at a concentration equal to the IC25 values for a given condition. Cells were treated for 24 h. The control was untreated ovarian cancer line cells. In addition, fibroblast cells, NHDF, were cultured. Exosomes were isolated from each culture after treatment and non-treatment control. Isolation of exosomes was performed using Total Exosome Isolation (from cell culture media) reagent (Invitrogen) from 10 mL samples of the medium. The isolation was carried out according to the producer’s instructions. Before treatment, cells were cultured for at least 24 h in an exosome-depleted FBS medium. Treatment and isolation were performed in triplicates.

### 2.6. Measurement and Visualization of Exosomes

The total protein content of vesicle lysates was estimated using a DS-11 FX Series Spectrophotometer/Fluorometer (DeNovix; Hanby Building, Wilmington, NC, USA).

Isolated exosomes, secreted by NHDF fibroblast cells, untreated A2780, TOV-21G, and SKOV-3 cells, treated with α-mangostin and/or cisplatin, were measured using the NTA technique (Nanoparticle analysis tracking) to visualize and analyze the size of suspended nanoparticles in liquid. The NTA measurement technique uses the Brownian motion of particles. Measurement was performed using the Malvern Panalytical NanoSight NS300 instrument (UK). The obtained results were statistically analyzed using NTA 3.2 Dev Build 3.2.16 software (Malvern, UK).

For further stages of the research, the visualization of exosomes was carried out using the dye VybrantTM CM-Dil (Invitrogen, Waltham, MA, USA) and microscopic analysis in the Nikon Eclipse Ti fluorescence microscope with the G-2A filter (λexc. = 510–500 nm; BA590 emission). VybrantTM CM-Dil is a lipophilic dye that diffuses into the membrane, staining exosomes orange-red. The exosomes were stained according to the ratio: 50 µL of exosomes: 1 µL of dye. Then it was incubated for 20 min at 37 degrees. Unincorporated dye was removed by gel filtration using Exosome Spin Columns (MW 3000) (Invitrogen, Waltham, MA, USA). Purification was performed according to the manufacturer’s instructions. PBS mixed with dye and purified on Exosome Spin Columns (MW 3000) was used as a control.

### 2.7. Exosome Uptake

The uptake of exosomes by fibroblast cells in in vitro culture was evaluated. NHDF cells were cultured on the chamber slide, 0.2 × 106 cells per well, in a medium supplemented with dFBS. Before treating fibroblasts with stained exosomes, cells were incubated in a medium with DAPI (Sigma-Aldrich, 150 nM, Saint Louis, MI, USA) at 37 °C for 20 min. After this time, cells were washed with PBS and treated with purified exosomes stained with VybrantTM CM-Dil (15 µg of exosomes in 500 µL of medium). Pictures were taken with the Nikon Eclipse Ti fluorescence microscope. Measurements were taken at 0, 3, 6, and 12 h after treatment of the cells. The control consisted of NHDF cells treated with stained PBS solution with VybrantTM CM-Dil.

### 2.8. Statistical Analysis

Results of the cell viability assay were analyzed statistically using the STATISTICA 13.1. (StatSoft, Warsaw, Poland). Significance was analyzed using ANOVA, followed by post hoc Tukey’s multiple comparison test. Data are presented as the mean ± standard deviation (SD). A *p* values less than 0.01 were considered statistically significant.

## 3. Results

### 3.1. The Results of the IC50 Value Determination

**Figure 1:**
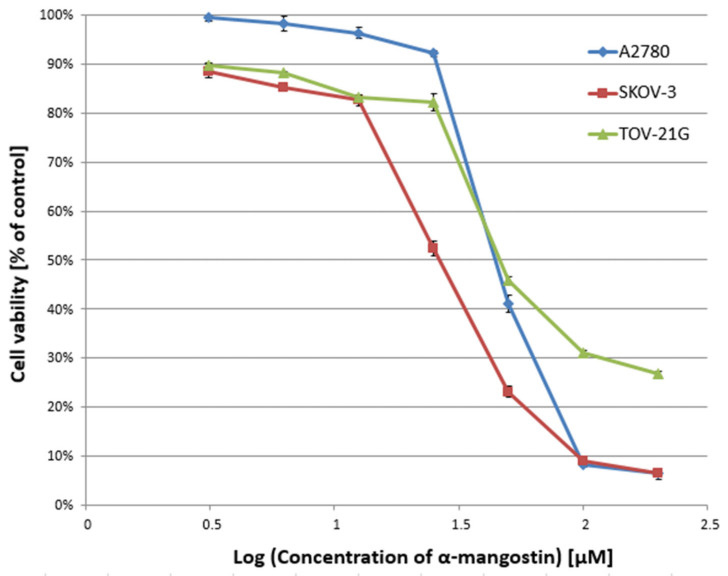
Effect of α-mangostin on the viability of A2780, SKOV-3, and TOV-21G human ovarian cancer cells measured by XTT assay and analyzed with ANOVA test. The cells were treated with indicated concentrations of modulators for 24 h. The data are shown as mean ± SD of triplicate experiments. All of the results were statistically significant (*p* < 0.001) between the cells treated with the different concentrations of modulators and the untreated control cells.

**Figure 2:**
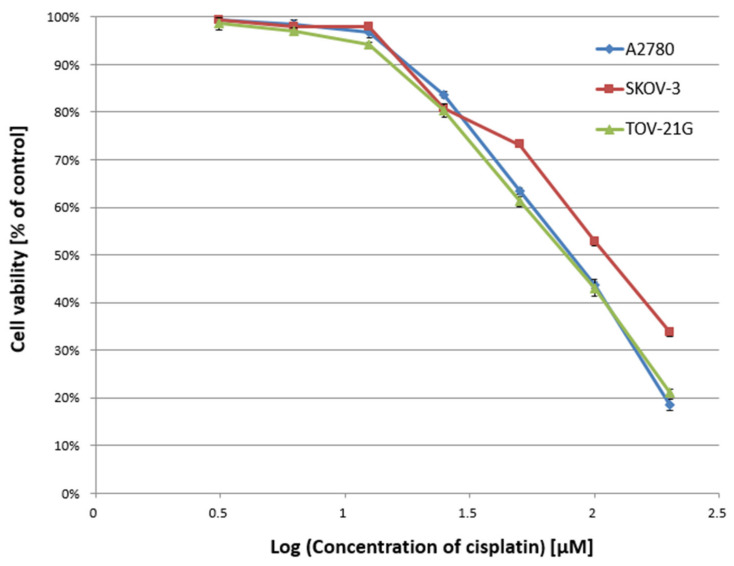
Effect of cisplatin on the viability of A2780, SKOV-3, and TOV-21G human ovarian cancer cells measured by XTT assay and analyzed with ANOVA test. The cells were treated with indicated concentrations of modulators for 24 h. The data are shown as mean ± SD of triplicate experiments. All of the results were statistically significant (*p* < 0.001) between the cells treated with the different concentrations of modulators and the untreated control cells.

In the first stage of the study, the cytotoxicity of α-mangostin (3.125–200 µM) and/or cisplatin (3.125–200 µM) was determined against A2780, SKOV-3, and TOV-21G cells for 24 h, and the IC50 value was determined. Figure 1 and Figure 2 show a summary of the obtained cytotoxicity results for individual cell lines. Inhibition of cell viability was dose-dependent for both α-mangostin and cisplatin. Table 1 shows the IC50 values determined for the individual cell lines and compounds. The determined IC50 values for cisplatin were higher for the SKOV-3 line (the resistance line according to ATCC cell line characteristics) compared to the other lines (for A2780 cells and TOV-21G, there was a statistical difference, *p* < 0.001). The determined IC50 values for mangostin were significantly lower than the IC50 values for cisplatin for each cell line tested. The most remarkable difference was observed for the SKOV-3 line (*p* < 0.01).

### 3.2. The Evaluation of the Combined Effect of α-Mangostin and Cisplatin on Cell Viability

**Figure 3:**
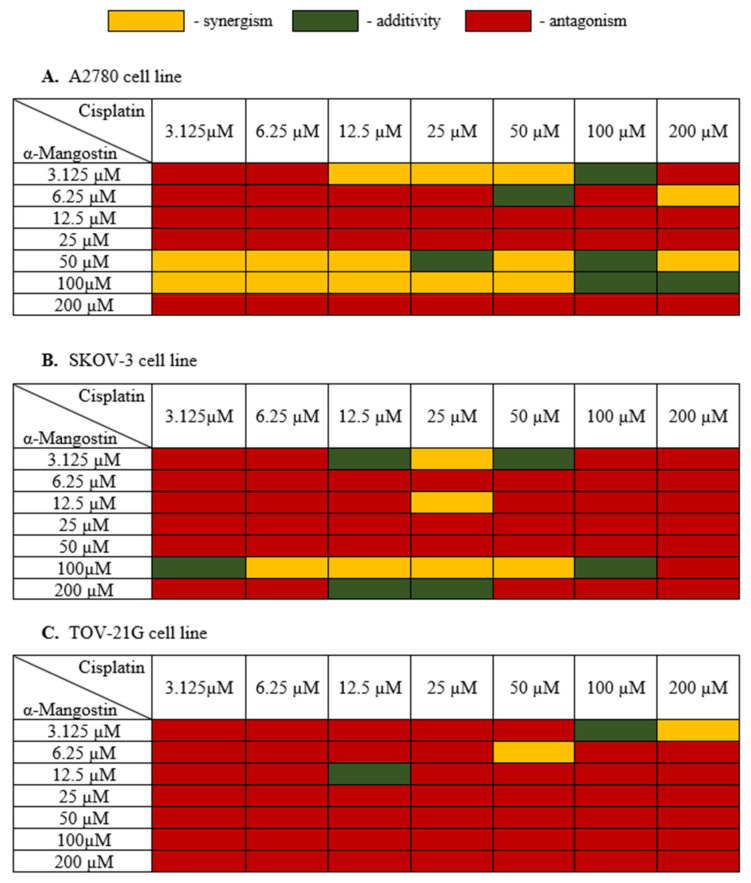
Cytotoxic effect α-mangostin—cisplatin combination in (**A**–**C**). After the XTT test was performed, the CI value was determined with CompuSyn (ComboSyn, Inc., Cambridge, MA, USA) and a heat map was generated. Yellow indicated a synergistic effect when the CI value was less than 0.9; green color when the effect of drugs was additive when the CI value was from 0.85 to 1.1; red color indicated an antagonistic effect when the CI value was above 1.1.

In the next stage, the cell viability of the combination of α-mangostin and cisplatin for a 24 h incubation time was carried out in order to assess the interaction between α-mangostin and cisplatin. The most effective doses of both drugs were selected for the next stages of the experiment. The measure of synergy between the two drugs, called the combination index (CI), was calculated using the CompuSyn software (ComboSyn, Inc., Cambridge, MA, USA) [39,40]. If the value of CI was below 0.9, the combination was synergistic; if the CI ranged from 0.9 to 1.1, the combination was additive; CI values above 1.1, the combination was antagonistic [40]. Figure 3 shows the obtained results of α-mangostin and cisplatin combinations for all three human ovarian cancer lines tested.

Treatment of A2780 cells with a combination of drugs with a high concentration of α-mangostin (200 µM) and its average concentrations (12.5 µM, 25 µM) together with cisplatin showed an antagonistic effect. Cisplatin showed a synergistic effect with α-mangostin when the concentration of α-mangostin was 50 µM or 100 µM. The additive effect of the compounds was observed mainly for high concentrations of cisplatin (100 µM) and α-mangostin (50 µM, 100 µM). The compounds showed mainly antagonistic activity at low doses. Based on the data described, the most advantageous combination of drugs were selected for further stages of the research as follows: 50 µM mangostin and 12.5 µM cisplatin. For this combination, A2780 cell viability was also considered, which was 31.0%.

The SKOV-3 cell line is a cisplatin-resistant human ovarian carcinoma line. The analysis of these cells’ treatment with both cisplatin and α-mangostin showed the antagonistic activity of the tested drugs in most cases, with the cell viability ranging from 42.31% to 99%. At a high concentration of α-mangostin (100 µM), the additive activity of the compounds was observed (for 3.125 µM and 100 µM of cisplatin), and a synergistic effect was observed in the range of 6.25–50 µM for cisplatin. An additive effect of the compounds was also observed in the case of a very low concentration of α-mangostin (3.125 µM) in combination with the average concentration of cisplatin (12.5 µM and 50 µM). The most favorable synergistic effect of α-mangostin and cisplatin was noted for 25 µM cisplatin in combination with 3.125 µM and 12.5 µM concentrations of α-mangostin. Based on the data described, the most favorable combination of drugs were selected for further stages of the research as follows: 12.5 µM of mangostin and 25 µM of cisplatin. The viability of SKOV-3 cells was also taken into account, which for this combination was 50.46%.

In most cases, the treatment of TOV-21G cells with α-mangostin and cisplatin showed an antagonistic effect of the investigated drugs. Synergistic activity was noted at high concentrations of cisplatin together with low doses of α-mangostin. An additive effect was observed in the following two combinations: 12.5 µM for both drugs and 100 µM for cisplatin with 3.125 µM for α-mangostin. Based on the obtained results, the most favorable combination of drugs for TOV-21G cells was selected for the following further steps: 50 µM cisplatin and 6.25 µM α-mangostin (the viability of cells was 61.23%).

### 3.3. Exosome Measurments—NTA Analysis Results

In the next step, the effect of α-mangostin and/or cisplatin on exosome release by human ovarian cancer cells was examined. A2780, TOV-21G, and SKOV-3 cells were treated with α-mangostin and cisplatin at a concentration equal to the determined IC25 value. Additionally, based on the results of the drug combination test, the most preferred α- mangostin and cisplatin combination was selected for each line. The following drug combination concentrations were selected: for line A2780: 50 µM α-mangostin and 12.5 µM cisplatin; for the TOV-21G line: 6.25 µM α-mangostin and 50 µM cisplatin and for the SKOV-3 line: 12.5 µM α-mangostin and 25 µM cisplatin. The human ovarian cancer cells were treated with selected concentrations of compounds. Then exosomes were isolated to obtain the following exosome samples: untreated A2780 cells (A2780 C), A2780 cells treated with α-mangostin (A2780 MA), A2780 cells treated with cisplatin (A2780 CIS), A2780 cells treated with α-mangostin and cisplatin (A2780 MA/CIS), untreated TOV-21G cells (TOV C), TOV-21G cells treated with α-mangostin (TOV MA), TOV-21G cells treated with cisplatin (TOV CIS), TOV-21G cells treated with α-mangostin and cisplatin (TOV MA/CIS), untreated SKOV-3 cells (SKOV C), SKOV-3 cells treated with α-mangostin (SKOV MA), SKOV-3 cells treated with cisplatin (SKOV CIS), SKOV-3 cells treated with α-mangostin and cisplatin (SKOV MA/CIS). Additionally, as a control, exosomes were isolated from untreated NHDF fibroblast cells (NHDF C). Each sample of the isolated exosomes was analyzed by NTA.

**Figure 4:**
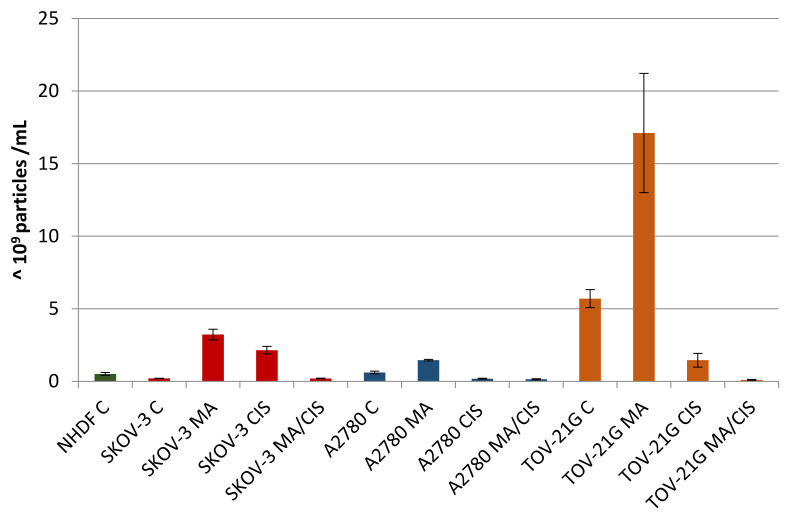
Number of exosomes (particles with a diameter of 30–150 nm) secreted by untreated NHDF, SKOV-3, A2780, and TOV-21G cells and SKOV-3, A2780, and TOV-21G cells treated with α-mangostin and/or cisplatin.

A comparative analysis of the number of secreted exosomes depending on their origin and treatment conditions was presented in Figure 4. Differences were observed both in individual cell lines after treatment with α-mangostin and/or cisplatin, as well as in relation to normal fibroblasts. For each examined ovarian cancer line, the secretion of the highest number of exosomes was observed after treatment with α-mangostin (in relation to the number of exosomes derived from untreated cells). A smaller number of exosomes were secreted after treatment with cisplatin. The lowest number of exosomes was observed after treatment with both α-mangostin and cisplatin.

The number of exosomes released varied depending on the origin and conditions of treatment. Treatment of A2780 cells with α-mangostin resulted in a twofold increase in the number of secreted exosomes compared to the untreated cells of this line. On the other hand, treatment with cisplatin alone and a mixture of α-mangostin and cisplatin contributed to a threefold and fourfold reduction in the number of particles released by these cells (at 31% and 24%, respectively).

The greatest differences in the number of secreted exosomes between untreated and treated cells were noted for cisplatin-resistant SKOV-3 cells. After treatment of SKOV-3 cells with both α-mangostin and cisplatin, the number of exosomes increased more than tenfold. The addition of α-mangostin increased the number of secreted exosomes by 15.6 times. The increase in the case of sole cisplatin was the lowest and equaled 10.4 times. On the other hand, when SKOV-3 cells were treated with the two drugs, no differences in the number of exosomes released were observed compared to untreated cells of this line.

TOV-21G cells released the largest number of exosomes among all the cell lines tested. When comparing the proportional changes in the number of released particles after treatment with compounds, a similarity to the cells of the A2780 line was observed. Treatment of TOV-21G cells increased the released exosomes by 200%. On the other hand, the treatment with cisplatin and simultaneous treatment with α-mangostin and cisplatin resulted in fewer exosomes being observed (at 26% and 2%, respectively).

The differences in the number of exosomes released by ovarian cancer cells compared to normal fibroblasts were significant. Untreated SKOV-3 cells secreted 60% fewer exosomes, while A2780 cells had 20% more particles and TOV-21G cells by more than 1000%. Regardless of the cell line tested, after the treatment of tumor cells with α-mangostin and cisplatin, the number of exosomes decreased, from 60% to 80%, in relation to the number of exosomes secreted by normal fibroblasts.

### 3.4. Results of the Uptake of Exosomes by Fibroblast Cells

**Figure 5:**
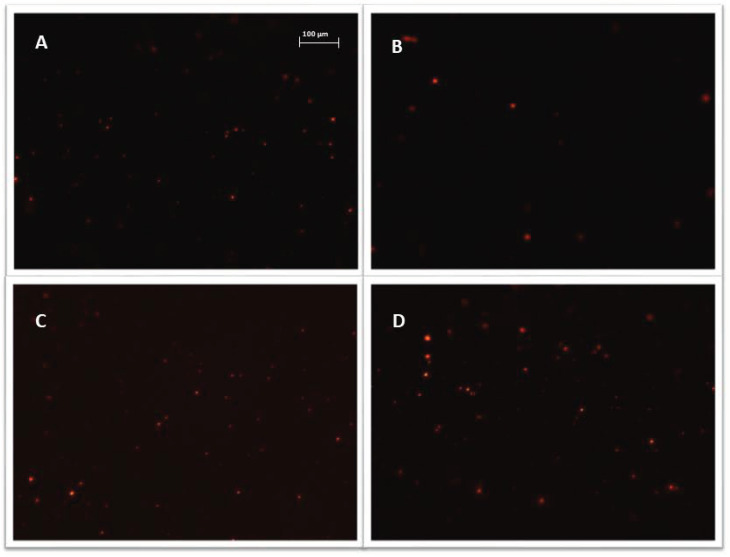
Representative images, exosomes (visible as red vesicles) stained with Vybrant ™ CM-Dil dye and purified on exosome spin columns (MW 3000), exosomes isolated from: (**A**) cisplatin treated A2780 cells; (**B**) α-mangostin treated TOV-21G cells; (**C**) cisplatin treated SKOV-3 cells; (**D**) untreated SKOV-3 cells. Nikon Eclipse Ti, magnification × 100.

All exosome samples were labeled with Vybrant™ CM-Dil and purified using exosome spin columns. Microscopic slides were used to verify the quality of the purified exosome samples. Figure 5 shows sample photos of stained exosomes.

**Figure 6:**
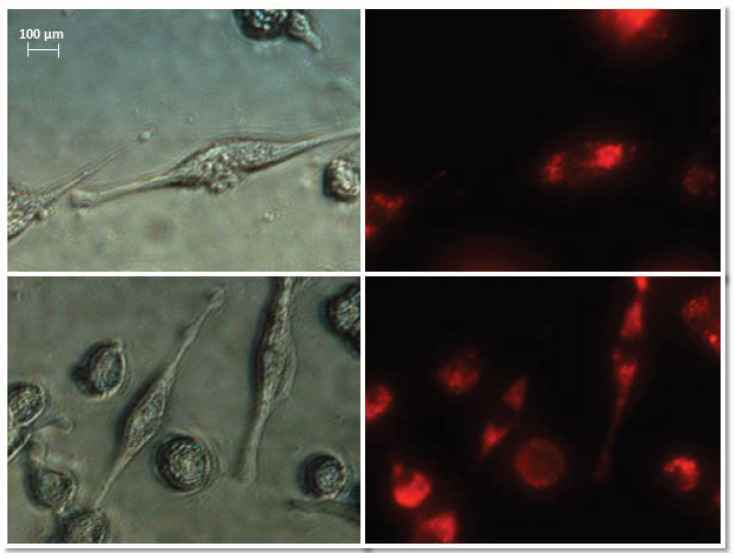
Representative images of NHDF cells (**left**, visible light) after treatment with exosomes (**right**, Vybrant ™ CM-Dil stained—red). Nikon Eclipse Ti, magnification × 40.

Normal fibroblasts were exposed to isolated, stained exosomes from ovarian cancer cells. Pictures were taken at 0, 3, 6, and 12 h after treating cells with exosomes. The analysis of the uptake of exosomes by fibroblasts showed differences in their activity depending on the origin. Sample photos of the distribution of exosomes inside the cell are shown in Figure 6.

**Figure 7:**
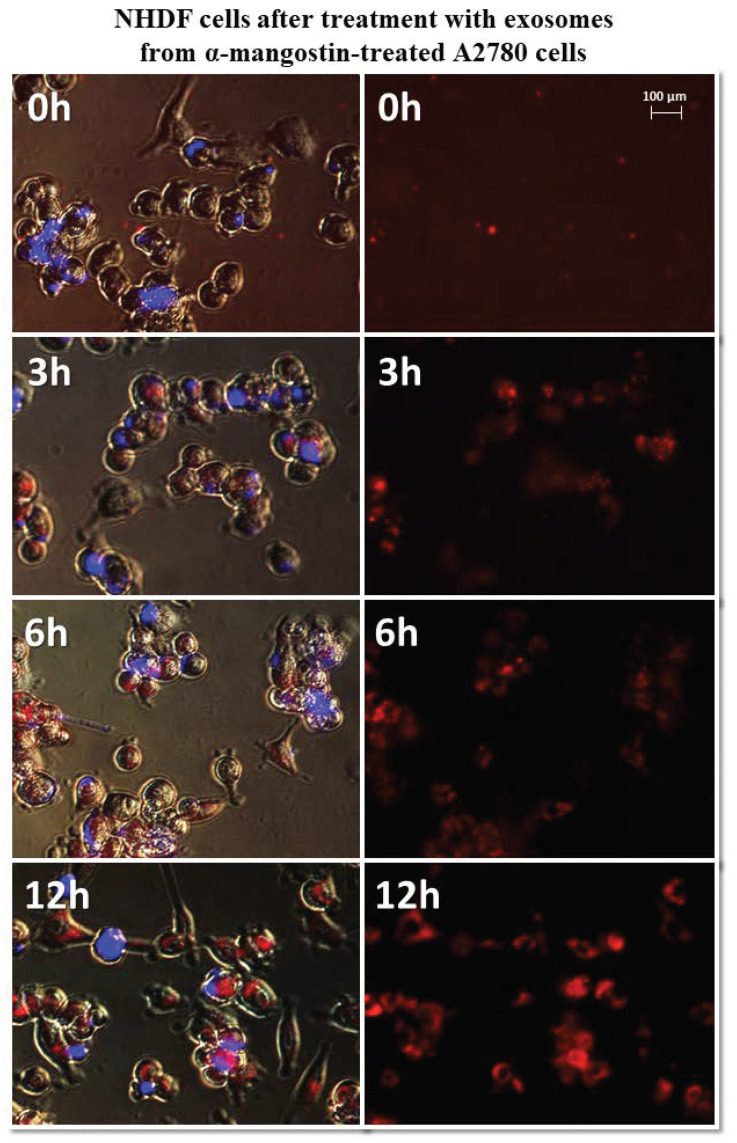
Representative photos of NHDF cells treated with exosomes derived from α-mangostin-treated A2780 cells at 0, 3, 6, and 12 h after treatment. Left: merged images of cells in visible light, DAPI stained cells (blue) and Vybrant™ CM-Dil stained exosomes (red), right: Vybrant ™ CM-Dil stained exosomes (red). Nikon Eclipse Ti, magnification × 20.

**Figure 8:**
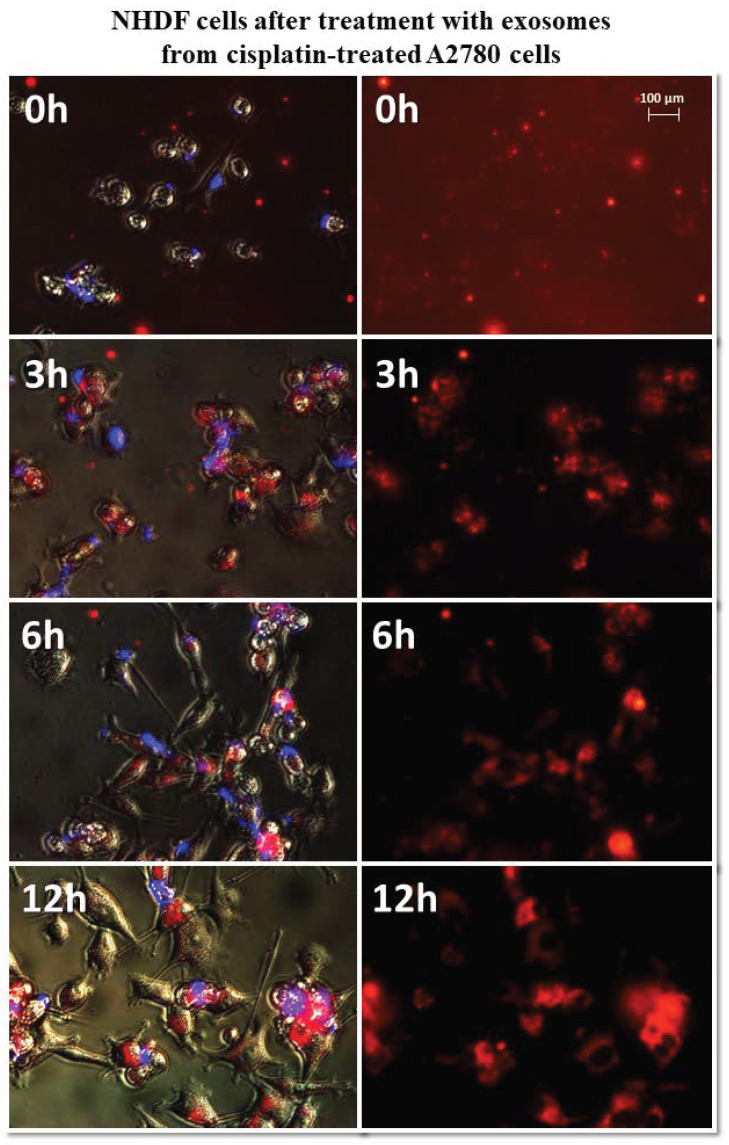
Representative photos of NHDF cells treated with exosomes derived from cisplatin-treated A2780 cells at 0, 3, 6, and 12 h after treatment. Left: merged images of cells in visible light, DAPI stained cells (blue) and Vybrant™ CM-Dil stained exosomes (red), right: Vybrant ™ CM-Dil stained exosomes (red). Nikon Eclipse Ti, magnification × 20.

It has been observed that exosomes derived from A2780 cells (regardless of the used drug or not) entered the fibroblasts after three hours and persisted inside the cells. Only in the case of exosomes isolated from A2780 cells after treatment with cisplatin (A2780 CIS) was delayed entry of exosomes into the cells was noted. Three hours after treatment, some of the exosomes had entered the cells. After six hours, free exosomes (except for cells) were not observed. Figure 7 and Figure 8 show examples of differences in exosome uptake depending on their origin by NHDF cells (A2780 MA and A2780 CIS). Twelve hours after treatment of fibroblasts, exosomes from untreated A2780 (A2780 C) cells partially left the cells. In other cases, exosomes isolated from A2780 cells persisted inside the cells.

The treatment of fibroblasts with exosomes derived from SKOV-3 cells (SKOV C, SKOV MA, SKOV CIS, SKOV MA/CIS) showed no significant observable differences. Regardless of the used SKOV-3 cells (drug-treated or not), uptake by NHDF cells after three hours was observed for most exosomes. The exosomes remained inside the cells for up to 12 h after treatment.

**Figure 9:**
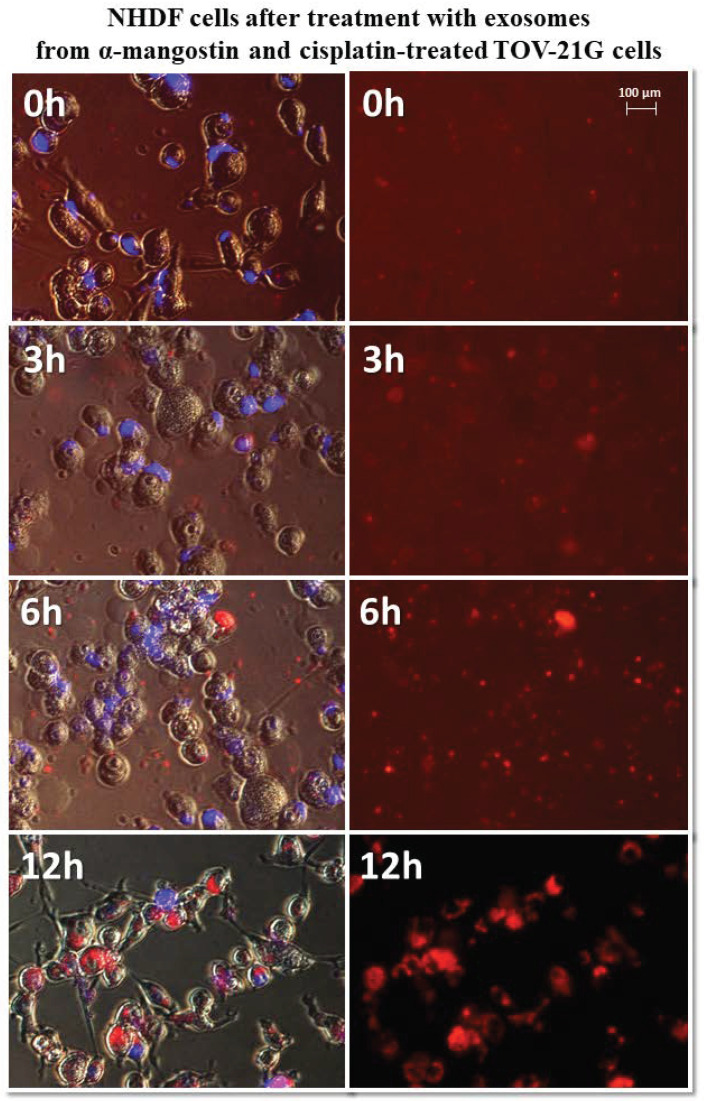
Representative photos of NHDF cells treated with exosomes derived from α-mangostin and cisplatin-treated TOV-21G cells at 0, 3, 6, and 12 h after treatment. Left: merged images of cells in visible light, DAPI stained cells (blue) and Vybrant™ CM-Dil stained exosomes (red), right: Vybrant ™ CM-Dil stained exosomes (red). Nikon Eclipse Ti, magnification × 20.

The greatest differences in exosome uptake were observed for exosomes derived from TOV-21G cells. The exosomes derived from untreated cells (TOV C) and α-mangostin-treated cells (TOV MA) entered the fibroblasts after three hours and persisted in the cells for up to 12 h. In the third hour after treatment with exosomes from cisplatin-treated cells (TOV CIS), partial uptake by the cells was observed. Only by the sixth hour after treatment, all were exosomes inside the cells. The biggest difference was noted for exosomes derived from cells treated with a combination of drugs as follows: α-mangostin and cisplatin (TOV MA/CIS). Within six hours of treatment with exosomes, most of them were outside the fibroblast cells. The complete uptake by cells was observed after 12 h. The exemplary images of the uptake of exosomes derived from TOV-21G cells treated with α-mangostin and cisplatin at 0, 3, 6, and 12 h were shown in Figure 9.

## 4. Discussion

Ovarian cancer is one of the deadliest cancers among women. Due to the lack of specific early symptoms of the development of this neoplasm and the relatively low specificity of screening tests, it is often detected at an advanced stage. In 90% of cases, it is epithelial ovarian cancer [41]. The current treatment protocols mainly include cytoreductive surgery and platinum-based chemotherapy, accounting for 80% of newly diagnosed patients. Unfortunately, patients with advanced ovarian cancer often suffer from a recurrence of the disease. In addition, cisplatin resistance in ovarian cancer poses a significant problem [42,43]. Due to the difficulties in both the diagnosis and treatment regimen, new and better alternatives for the treatment of ovarian cancer are still being sought. The use of natural substances has been discussed and researched for many years. One of the branches of this research is xanthones. The most advanced research among this broad group of naturally occurring compounds was conducted on α-mangostin [44] and gambogic acid [45].

This study focuses on the possibility of using α-mangostin alone or in combination with cisplatin, currently used in ovarian cancer chemotherapy. α-Mangostin has a broad anticancer effect, inter alia, in colorectal cancer [46], lung cancer [47], kidney cancer [48], breast cancer [49], squamous cell carcinoma of the head and neck [50], and ovarian cancer [51].

The studies were conducted on three ovarian cancer lines, including one cisplatin-resistant SKOV-3 cell line. For the first time, an attempt was made to evaluate the combined effect of α-mangostin and cisplatin on ovarian cancer cells. Studies have shown that these drugs together in the range of specific concentrations show a synergistic effect, but it depends on the type of cell line tested. It was observed that the same cytotoxic effect with cisplatin treatment alone is possible with the simultaneous treatment of the cells with α-mangostin and cisplatin at a lower concentration. The use of cisplatin is associated with many side effects. The accumulation of cisplatin in various organs leads mainly to nephrotoxicity, hepatotoxicity, neurotoxicity, and cardiotoxicity [52]. In the case of A2780 cells treated with cisplatin, the IC50 value was 80.25 µM, while the same cytotoxic effect was obtained when cells were treated with α-mangostin and cisplatin together at a cisplatin concentration of 12.5 µM. For cisplatin-treated TOV-21G cells, the IC50 value was 77.56 µM, while the same cytotoxic effect was obtained by treating the cells with the two drugs at a cisplatin concentration of 50 µM. A synergistic effect of α-mangostin and cisplatin was also observed on cisplatin-resistant SKOV-3 cells. The IC50 value for those cells treated with cisplatin alone was 111.06 µM, while the treatment of cells with the combination of α-mangostin and cisplatin at a concentration of 25 µM. The obtained results indicate that the use of mangostin in combination with cisplatin may contribute to the possibility of lowering the cisplatin concentration while maintaining the same cytotoxic effect. There is little research on the effects of α-mangostin on ovarian cancer cells. Such studies were carried out by Yu et al., who conducted their research on the ovarian cancer cell line OVACAR-3. They showed that α-mangostin induces antiproliferative activity, enhances the apoptosis process, inhibits the m-TOR/PI3K/AKT signaling pathway, and increases the production of free radicals [51]. The cisplatin’s primary mechanism of action is based on drug-DNA interactions and the formation of DNA adducts, including mono-, inter-, and intrastrand cisplatin DNA cross-links. This leads to cell cycle arrest and the induction of apoptosis [53].

The fact that both α-mangostin and cisplatin induce apoptosis in cancer cells by different mechanisms gives hope for the possibility of combining these substances in chemotherapy, thus reducing side effects. Studies on rats have already shown the cardioprotective effect of α-mangostin on doxorubicin-induced cardiotoxicity (used against a wide range of malignancies including breast cancer, sarcoma, Hodgkin’s disease, non-Hodgkin’s lymphoma, and acute leukemia) [54]. Another example is the synergistic effect of 5-fluorouracil (5-FU) with α-mangostin. The combination of these drugs inhibited the growth of DLD-1 colorectal cancer cells, reducing the clinical dose of 5-FU and thereby reducing the systemic cytotoxicity of 5-FU [55].

The studies were carried out on three different cell lines of ovarian cancer. Epithelial ovarian cancer (EOC) is the most common type. In our research, represented by the A2780 line. It is characterized by both the ease of relapse and the ease of metastasis. Hence, the high mortality among women suffering from EOC [56]. The main cause of EOC development is hereditary mutations. They often involve mutations in BRCA1 or BRCA2 or, to a lesser extent, mutations in RAD51C, RAD51D, PALB2, and FANCI. Most EOC tumors are also characterized by a p53 mutation [57]. Clear cell carcinoma (CCC) of the ovary is a less common type of ovarian cancer, and the TOV-21G line represents it in our research. Clear cell carcinoma is strongly associated with the incidence of endometriosis. Endometriosis is believed to be the direct cause of this type of ovarian cancer. However, it is associated with a good prognosis [58]. The most common mutations in CCC are mutations in the ARID1A gene and the PIK3CA gene [59]. The last cell line tested was SKOV-3. Cells of this line represent adenocarcinoma of the ovary. In our research, SKOV-3 cells were cisplatin-resistant. The above-described differences between the tested lines indicate a considerable variation among ovarian cancer cells. Hence, our results were distinctive for each tested cell line. Despite such a large variety, we observed that the joint treatment of cells with cisplatin and α-mangostin allows the reduction of the cisplatin dose. Ovarian cancer is not the only cancer for which new drugs are being sought. New independent substances with anticancer activity are sought, which could be combined with currently used drugs [60,61,62].

Ovarian cancer cells can regulate immune activation and suppression by presenting tumor antigens to immune cells or by secreting cytokines. The microenvironment modulation also occurs by the exosomes secreted by these cells. Exosomes derived from ovarian cancer cells actively carry biological molecules, including the histocompatibility complex (MHC I), heat shock protein (HSP), and CD81 [63]. Many studies indicate a significant role of exosomes in ovarian cancer metastasis. In the conducted research, we tried to answer the question of whether the use of drugs like α-mangostin and/or cisplatin affects the number of exosomes released by ovarian cancer cells. Additionally, we have made an initial attempt to answer the question of whether exosomes isolated from treated ovarian cancer cells affect the microenvironment—normal fibroblasts. The process of ovarian cancer metastasis to the peritoneum begins with the detachment of cells from the primary site (ovary or fallopian tube). The cancer cells then spread into the peritoneal cavity and attach to the organ surfaces of the peritoneum, particularly the network, which is the primary site for ovarian cancer metastasis. All organs in the peritoneal cavity are covered with a single layer of mesothelial cells with an underlying stroma. In the metastatic process, detached ovarian cancer cells first attack the mesothelial barrier [64]. Both primary tumor cells and detached neoplastic cells secrete exosomes into the environment, thus affecting the cells of the immune system (T-cells, NK-cells, macrophages), fibroblasts, peritoneal mesenchymal cells, and endothelial cells [64].

Our research tested the following three different ovarian cancer cell lines: A2780 (epithelial ovarian cancer cell line), cisplatin-resistant SKOV-3, and TOV-21G (grade 3, stage III, primary malignant adenocarcinoma; clear cell carcinoma). Among three of the analyzed cell lines, the cisplatin-resistant SKOV-3 cell line released the smallest number of exosomes, while the cells of advanced follicular carcinoma TOV-21G released significantly more. Interestingly, in all cases, a significant increase in the number of exosomes was observed after the treatment with α-mangostin. The observed variability of secreted exosomes was caused by both the origin of the tumor cells themselves and the different therapeutic approaches (using α-mangostin and/or cisplatin). This indicates the great individuality of cancer itself and the need for personalized treatment. For each examined ovarian cancer line, the secretion of the highest number of exosomes was observed after treatment with α-mangostin (in relation to the number of exosomes derived from untreated cells). A smaller number of exosomes were secreted after treatment with cisplatin. The lowest exosomes were observed after treatment with both α-mangostin and cisplatin. Perhaps the protective effect of α-mangostin, described in the literature in cells treated with the simultaneous action of cisplatin [37,65], also translates into a reduction in the number of exosomes secreted by these cells.

Exomes make it possible to use them as potential drug carriers. Drugs can enter the exosomes based on the difference in concentration gradients. However, this is dependent on the hydrophobicity of the drug used. The treatment of target cells with drug-loaded exosomes may induce a therapeutic effect [66]. This seemingly simple mechanism is supposed to allow the use of exosomes as drugs. Most of the current research focuses on using exosomes as drug carriers. However, it will certainly be a long time before we come to validate the full use of exosomes in current chemotherapy. Our research attempted to assess whether the use of drugs affects both the cancer cells themselves and the secretion of exosomes. At the same time, to verify whether exosomes secreted from tumor cells (after drug stimulation) show a different effect on normal fibroblasts. This work shows that most exosomes enter and persist in fibroblasts after three hours. In our further work, we will check how these exosomes interact with cells at the molecular level. The cisplatin-treated cells of the A2780 and TOV-21G lines observed delayed uptake of exosomes by fibroblasts only after six hours. Interestingly, TOV-21G cells treated with α-mangostin and cisplatin released the smallest number of exosomes into the microenvironment. At the same time, poor uptake of these exosomes by fibroblasts was observed. All of the exosomes migrated into the cells after 12 h. The rate of entry of exosomes into cells may affect metastasis, but the load carried by exosomes is critical. It is known that neoplastic cells after treatment with drugs, both α-mangostin and cisplatin, change at the molecular level [53,67]. Presumably, this altered load is carried along with the exosomes released from these cells. Hence, there were significant differences in the number of molecules released after the treatment of tumor cells with drugs.

## 5. Conclusions

Ovarian cancer is a very heterogeneous tumor. In this study, we showed that treated A2780, SKOV-3, and TOV-21G cells with α-mangostin in combination with cisplatin might reduce the concentration of cisplatin while maintaining the same cytotoxicity effect. Our research indicates the possibility of using α-mangostin as a potential candidate in oncological therapy combined with cisplatin. Numerous studies indicate that this may be associated with fewer toxic effects caused by cisplatin itself. One aspect that can be used in therapy is the assessment of the chemotherapeutic effect on cancer cells. The second important aspect is the evaluation of the influence of chemotherapeutic agents on the possibility of modulating the microenvironment. The number of exosomes released by ovarian cancer cells in different stages and treatment conditions was assessed in this context. Under the influence of α-mangostin and/or cisplatin, ovarian cancer cells release a variable number of exosomes into the microenvironment. For each examined ovarian cancer line, the secretion of the highest number of exosomes was observed after treatment with α-mangostin (in relation to the number of exosomes derived from untreated cells). A smaller number of exosomes were secreted after treatment with cisplatin. The lowest number of exosomes was observed after treatment with both α-mangostin and cisplatin. It is necessary to conduct further research on whether the protective effect of α-mangostin described in the literature in cells subjected to the simultaneous action of cisplatin [44,65] translates into a decrease in the number of exosomes secreted by these cells. At the same time, we have already undertaken further studies at the molecular level to accurately confirm the role of exosomes in the ovarian cancer microenvironment. In particular, considering the variability of the exosome load after treating tumor cells with α-mangostin, cisplatin, or both.

## Figures and Tables

**Table 1:** IC50 value [µM] for α-mangostin and cisplatin against A2780, SKOV-3, and TOV-21G cells for 24 h incubation.

Cell Line/Compound	α-Mangostin	Cisplatin
A2780	47.74 ± 1.50	80.25 ± 5.00
SKOV-3	25.03 ± 0.50	111.06 ± 6.52
TOV-21G	59.96 ± 0.25	77.56 ± 1.89

## Data Availability

Data are available on request from the authors.
